# Unveiling Hidden Hyperuniformity: Radial Turing Pattern Formation of Marangoni‐Driven SiO_2_ Nanoparticles on Liquid Metal Surface

**DOI:** 10.1002/advs.202400163

**Published:** 2024-07-29

**Authors:** Jinjian Guo, Jie Chen, Kang Zhao, Xuedong Bai, Wenlong Wang

**Affiliations:** ^1^ State Key Laboratory for Surface Physics Beijing National Laboratory for Condensed Matter Physics Institute of Physics Chinese Academy of Sciences Beijing 100190 China; ^2^ Key Laboratory of Magnetic Molecules and Magnetic Information Materials of Ministry of Education Shanxi Normal University Taiyuan 030000 China; ^3^ School of Physical Sciences University of Chinese Academy of Sciences Chinese Academy of Sciences Beijing 100190 China; ^4^ School of Electronic and Information Engineering Tiangong University Tianjin 300387 China; ^5^ MOE Key Laboratory of Bioinformatics Beijing Advanced Innovation Center for Structural Biology and Frontier Research Center for Biological Structure Center for Synthetic and Systems Biology School of Life Sciences Tsinghua University Beijing 100190 China; ^6^ Songshan Lake Materials Laboratory Dongguan Guangdong 523808 China

**Keywords:** brownian motion, extreme environment, nanomotors, pattern formation, self‐Marangoni effect

## Abstract

Mastering the self‐organization of nanoparticle morphologies is pivotal in soft matter physics and film growth. Silicon dioxide (SiO_2_) nanoparticles are an archetypical model of nanomotor in soft matter. Here, the emphasis is on the self‐organizing behavior of SiO_2_ nanoparticles under extreme conditions. It is unveiled that manipulating the states of the metal substrate profoundly dictates the motion characteristics of SiO_2_ nanoparticles. This manipulation triggers the emergence of intricate morphologies and distinctive patterns. Employing a reaction‐diffusion model, the fundamental roles played by Brownian motion and Marangoni‐driven motion in shaping fractal structures and radial Turing patterns are demonstrated, respectively. Notably, these radial Turing patterns showcase hyperuniform order, challenging conventional notions of film morphology. These discoveries pave the way for crafting non‐equilibrium morphological materials, poised with the potential for self‐healing, adaptability, and innovative applications.

## Graphical abstract

Investigating the collective behavior of SiO_2_ nanoparticles on liquid metal surfaces reveals the complex SiO_2_ patterns formation driven by Marangoni flow. Manipulating metal substrate states influences particle motion, leading to fractal structures and radial Turing patterns. Hyperuniform order challenges conventional film morphology understanding. It is expected to be used to create “hyperuniform” smart fluid materials.

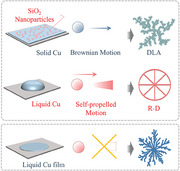

## 1 Introduction

Morphology management has attracted great interest in particle aggregates^[^ ^1^ ^]^ or thin film growth,^[^ ^2^ ^]^ as microstructure profoundly influences material properties such as optical, magnetic, electrical, and catalysis.^[^ ^3^ ^]^ In 2D thin film material growth, the Wulff construction^[^ ^4^ ^]^ is the equilibrium morphology of these 2D crystals in thermodynamic equilibrium, arising from the minimum Gibbs free energy or the reaction‐limitation controlled. For instance,^[^ ^4^, ^5^ ^]^ graphene adopts a hexagonal shape, while diatomic components h‐BN and MoS_2_ exhibit triangular equilibrium morphologies. Far from thermodynamic equilibrium, the film morphology would transform into a dendritic fractal structure due to diffusion‐limited growth control.^[^ ^6^ ^]^ As yet, Wulff construction and dendritic fractals dominate film morphology studies,^[^ ^6^, ^7^ ^]^ with exploration of more complex morphologies further away from equilibrium remaining rare and challenging. These complexities offer avenues for novel physical properties; for example, materials with intricate structures like Sierpinski triangles or Mandelbrot set patterns hold promise for emerging fields like topological fractal photonics, as highlighted by Lumer^[^ ^8^ ^]^ and Norris.^[^ ^9^ ^]^ Essentially, Wulff construction and fractal morphologies stem from Brownian motion‐driven particle collisions and bonding at growth fronts.^[^ ^2^ ^]^ If the particles at the growth front can be dynamically self‐assembled,^[^ ^10^ ^]^ transforming from the traditional surface growth mode to the study of dynamic systems and complex pattern formation, may unveil new morphology thin film materials, new physical properties, and new applications.

Dynamically self‐assembled particles^[^ ^10^ ^]^ that harvest environmental energy and lead to ordered structures or patterns have made remarkable progress over the last two decades. Numerous research groups^[^ ^11^ ^]^ have employed micron self‐propelled particles to generate various dynamically ordered structures such as aster, spiral, vortex, and antivortex. Unlike ordinary Brownian motion particles, these particles are active matter with self‐propelled motion. They can extract energy from their environment into mechanical work to drive their motion while interacting with each other to form collective behaviors (most commonly through fluids).^[^ ^11a^ ^]^ Despite the complexity of collective motion, all agents share some common characteristics, including self‐assembly, self‐organization, and far‐from‐equilibrium reaction‐diffusion processes.^[^ ^10^ ^]^ Notably, Ni Ran et.al ^[^ ^12^ ^]^ recently unveiled a new active fluid state in the collective behavior of self‐propelled particles: the hyperuniform and local giant fluctuations could coexist. It suggested the potential for smart active fluid materials with “self‐healing” and “adaptive”. Therefore, the autonomous movement and interaction features of self‐propelled particles have great potential in creating complex thin film morphologies and smart materials.

Here, we employ the liquid metal dynamic self‐assembly strategy and challenge the problem of controlling the complex morphology of thin film far from equilibrium. We focus on the aggregation and growth of SiO_2_ nanoparticles on the metal substrates at high temperatures, serving as a model system. Through manipulation of the solid and liquid physical properties of the metal substrate, we successfully controlled the novel complex morphology formation of SiO_2_ nanoparticles far from equilibrium, transitioning from fractal growth on the solid Copper (Cu) surface to the radial Turing (spoke) pattern formation on the liquid Cu surface (with size ≈1 µm). Notably, the SiO_2_ nanoparticles on the liquid metal surface behave as the Marangoni swimmer^[^ ^13^ ^]^ or Marangoni‐driven engine. Because oxides are efficient surfactants for liquid metal,^[^ ^14^ ^]^ the SiO_2_ nanoparticles could produce self‐propelled motion under the gradient of surface tension or the Marangoni effect (see Figure S1, Supporting Information for detailed discussion). Meanwhile, the liquid metal with giant surface tension provides the possibility for studying the novel collective behavior of self‐propelled nanoparticles in extreme environments. As expected, liquid Cu enables SiO_2_ nanoparticles to exhibit the coexistence of hyperuniform and giant fluctuations (i.e., the Spoke pattern is filled with maximally random jammed packing), validating previous theoretical predictions.^[^ ^12^ ^]^ The simulation of Diffusion‐limited‐aggregation (DLA) and Mimura‘s reaction‐diffusion model revealed that the morphology evolution arises from the motion properties of SiO_2_ nanoparticles, transitioning from Brownian to self‐propelled. SiO_2_ serves as a common research subject in both self‐propelled particles and the growth of low‐dimensional functional films. This work is the deep cross‐integration of these two fields, offering a new model system for soft matter physics and facilitating the discovery of new physical phenomena. Moreover, it provides strong support for thin film technology development and promotes the discovery of novel physical properties.

## 2 Results and Discusion

**Figure 1:**
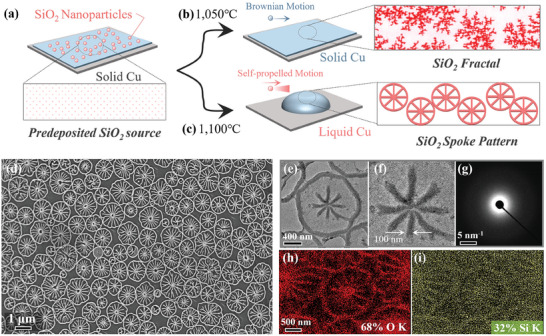
Morphology management of SiO_2_ nanoparticles at different interfaces. a–c) Schematic illustration of the SiO_2_ nanoparticle self‐organization on the solid and liquid Cu surface. d) SEM image of spoke patterns. e,f) TEM image of the pattern. g) SAED pattern of spoke pattern. h,i) SEM‐EDS mapping of pattern, O (red), and Si (green).

**Figures** **1**a and S2 (Supporting Information) are schematic diagrams of the experimental design of this work. Some SiO_2_ nanoparticles are pre‐deposited on the surface of the Cu substrate through low‐pressure chemical vapor deposition (LPCVD) (Figure 1a; Figure S4a, Supporting Information). Then, a vacuum–liquid metal Cu gas–liquid interface is created by raising the temperature of the solid Cu substrate above the melting point (see experiment details and Figure S3, Supporting Information), moving the system away from equilibrium and producing conditions for the emergence of novel non‐equilibrium film morphologies. On the solid Cu substrate, SiO_2_ nanoparticles self‐organize to form a dendritic fractal structure (Figure 1b; Figure S4b, Supporting Information). Remarkably, on the liquid Cu substrate, a novel morphology of flower‐like radial spoke patterns emerges (Figure 1c). Figure 1d shows the scanning electron microscope (SEM) image of this novel pattern, it exhibits fine and periodic stripes of about tens of nm in width, and the pattern size is ≈1 µm. To the best of our knowledge, similar patterns have been observed only in two other systems: the particle system of the coffee ring,^[^ ^15^ ^]^ and the bacterial colony pattern.^[^ ^16^ ^]^ However, compared with the prior system, the pattern of this system is different or unusual in two aspects. First, the scale of the patterns is significantly smaller. The previous similar morphology occurred on a centimeter scale, but this system's pattern appears on the nanoscale. Second, the patterns appear in groups. Previous systems generated individual patterns, whereas, in our system, patterns emerged in groups.

To determine the composition and structure of the aggregated morphology, electron microscopy analysis was conducted using the Spoke pattern as an example (Figure 1d,i). To begin, the sample of the spoke pattern on the copper surface was transferred onto a transmission electron microscope (TEM) grid by wet chemical methods^[^ ^17^ ^]^ (Figure S5, Supporting Information). From the TEM test (Figure 1e,f), the pattern size is 1.2 µm and the line width is 100 nm. Furthermore, the pattern is dense, indicating that there exists a particle fusion or particle coarsening process^[^ ^18^ ^]^ during pattern formation. Then, the amorphous nature of SiO_2_ was confirmed by the selected area electron diffraction pattern (SAED), as seen in Figure 1g. The sample's diffraction pattern exhibited a halo, comparable to the prior diffraction halo of amorphous SiO_2_ materials.^[^ ^19^ ^]^ Finally, we used energy dispersive spectroscopy (EDS) to estimate the atomic composition and atomic percentage of the sample. The SEM‐EDS mappings as depicted in Figure 1h,i, show that the sample comprises solely Si (blue) and O (red) elements. The SEM and TEM EDS atomic percentages testing of Si and O are 68:32 and 68.92:31.08 (Figure S6, Supporting Information), respectively, indicating that the sample is close to the stoichiometric ratio of SiO_2_ (2:1). To summarize, these aggregated structures of nanoparticles are composed of uniform and dense amorphous SiO_2_.

**Figure 2:**
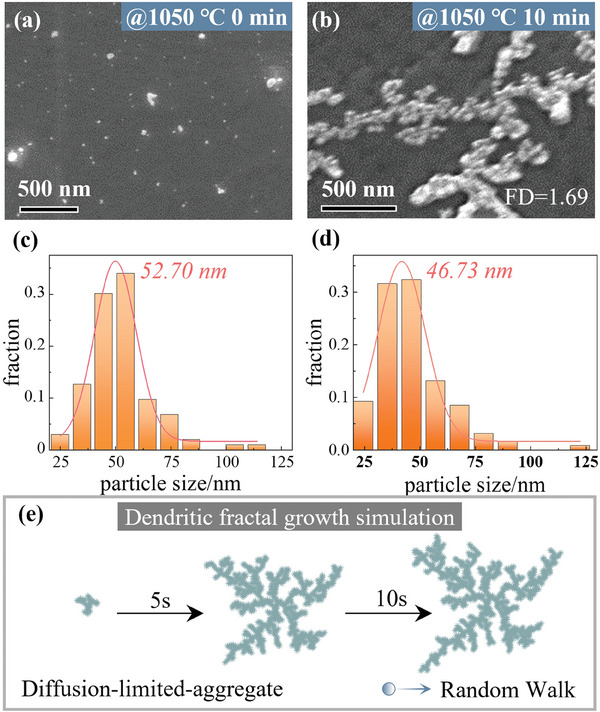
SiO_2_ nanoparticles self‐organize to form the fractal structure on a solid Copper substrate and its morphology simulation. a,b) The SEM images of uniform predeposited SiO_2_ source and dendritic fractal aggregate morphology on the solid Cu surface. c,d) The particle sizes distribution in panels (a, b) with Gaussian fitting. e) Numerical simulation of DLA fractal aggregation growth in MATLAB.

**Figure** **2**a,b depicts the SEM images of predeposited SiO_2_ nanoparticles and SiO_2_ fractal aggregate morphology on a solid Cu substrate when heated at 1050 °C for 0 and 10 min (Cu melting point 1083 °C), respectively. Subsequently, we investigated the particle size distribution to ascertain that the fractal aggregates originate from these pre‐deposited SiO_2_ nanoparticles, as the in situ aggregation process cannot be observed. The results are shown in Figure 2c,d, both of which have broad Gaussian distributions centered around a size of ≈50 nm, indicating that the SiO_2_ fractal structure indeed evolves from the source particles. Furthermore, the constituent particles of the fractal aggregates on the solid substrate are loose and simply sit next to each other without particle fusion or coarsening.

Then, we further measured the fractal dimension (FD) of the SiO_2_ fractal structure is 1.69 ± 0.05 (Figure S7, Supporting Information), which is rather close to the value for the classical DLA model (FD = 1.71),^[^ ^20^ ^]^ illustrating that the fractal aggregation morphology of SiO_2_ nanoparticles may satisfy DLA dynamics. Therefore, to reproduce and verify the formation mechanism of fractal morphology, we simulated the classical DLA model fractal growth of particles in Matlab. The simulation process is as follows: First, a seed is defined in the center of the square region, and then the particles approach the seed from the edge in Brownian motion (random walk). When they collide with and attach to the seeds (i.e., hit and stick, reaction probability (P) is 100%), particle clusters are formed; this process is repeated until a dendritic condensate is formed in the center of the square region. The simulation results are shown in Figure 2e, which are highly consistent with the experimental results of the SiO_2_ nanoparticle's fractal morphology (Figure 2b), indicating that the fractal morphology on the solid substrate is the result of the diffusion‐limited process. More importantly, it also reflects that the motion characteristics of SiO_2_ nanoparticles on the solid substrate obey Brownian motion.

**Figure 3:**
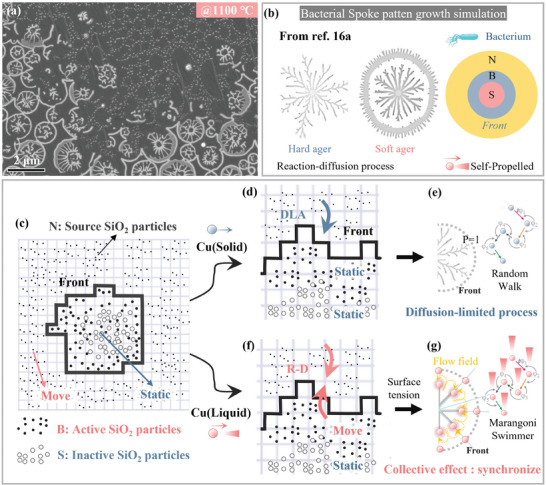
SiO_2_ nanoparticles self‐organize to form the radial Turing pattern on the liquid Copper substrate and its formation mechanism. a) SEM image of spoke pattern formation on the liquid metal surface during the intermediate process. b) DLA‐like fractal structure and Spoke patch diagram in bacterial pattern and modeling schematic diagram of Mimura's reaction‐diffusion model simulation, taken from ref. [16a]. c–g) The phenomenological models illustrate the mechanism for the emergence of the SiO_2_ fractal pattern and SiO_2_ spoke pattern.

**Figure** **3**a shows a frozen SEM image of the intermediate process of nanoparticles self‐organizing to form a spoke pattern on the liquid metal surface at 1100 °C. A key question then arises, why does the morphology change from a fractal structure (solid substrate) to a spoke pattern (liquid substrate)? A similar pattern evolution phenomenon was also observed in bacterial pattern experiments, as previously described. As shown in Figure 3b, bacteria grew into DLA‐like and spoke patterns on hard or soft substrates respectively, which showed high consistency with our work. Moreover, bacteria are typical self‐propelled active particles and SiO_2_ nanoparticles on the liquid Cu surface are also active particles by Marangoni‐driven motion as mentioned above, both have driving‐dissipation processes.^[^ ^10^, ^21^ ^]^ Therefore, the existing bacterial pattern model would inspire and help this work to solve the key question of morphological evolution. Taking the Mimura reaction‐diffusion model^[^ ^16^ ^]^ as an example, bacteria or active particles are divided into the following three categories according to their functions:

(1) $$\frac{\partial B}{\partial t}=D_{B}\;\nabla^{2}B+\varepsilon BN-\frac{\mu B}{\left(B+1\right)\left(N+1\right)}$$

active bacterial change = diffusion + reproduction − conversion

(2) $$\frac{\partial N}{\partial t}=\nabla^{2}\;N-NB$$

nutrient change = diffusion − conversion

(3) $$\frac{\partial S}{\partial t}=\frac{\mu B}{\left(B+1\right)\left(N+1\right)}\;$$

inactive bacterial change = conversion

As illustrated in Figure 3b, the model defines the bacteria at the growth front as active bacteria (B); Active bacteria (B) consume nutrients (N) for propagation, and as the growth front advances, they transform into inert bacteria (S). Equations ((1), (2), (3)) depicts the reaction‐diffusion process of B, N, and S. The author^[^ ^16a^ ^]^ successfully controlled the reaction‐diffusion process of bacteria and realized the evolution between the DLA fractal and Spoke pattern by adjusting the experimental parameters nutrient initial concentration (n_0_), and the substrate softness and hardness (d). Clearly, DLA is an extreme case in the reaction‐diffusion equation. Based on this knowledge, we proposed a phenomenological model of SiO_2_ nanoparticle Spoke pattern formation and compared it to the classic DLA formation process. As seen in Figure 3c–g, SiO_2_ nanoparticles are also classified into three different categories based on their functions: tiny particles (N), particles at the growth front (B), and inert particles after the front (S). On the solid substrate (Figure 3d), B and S cannot move actively and only wait for N Brownian motion to adhere (Figure 3e). Conversely, on the liquid substrate (Figure 3f), N is Marangoni swimmers, causing intense collisions and dissipation. B at the front can actively move, generating numerous interactions or collective/synergistic effects through the surface tension gradient and Marangoni flow. Those complex interactions of making particles move toward the center, repel, or attract each other, ultimately lead to the emergence of a new order structure S (Figure 3g). In the bacterial pattern, packaging these complex interactions in the nonlinear terms of the equations, and solving the equations, revealed that the collective emergence of this radial spoke pattern originates from the Turing instability.^[^ ^16c^ ^]^ Notably, the Spoke pattern is a variant of the well‐known Turing pattern.^[^ ^22^ ^]^ Therefore, the surface tension gradient created by liquid metal significantly enhances the complexity of the reaction‐transport process of SiO_2_ nanoparticles, resulting in the formation of novel order structures.

**Figure 4:**
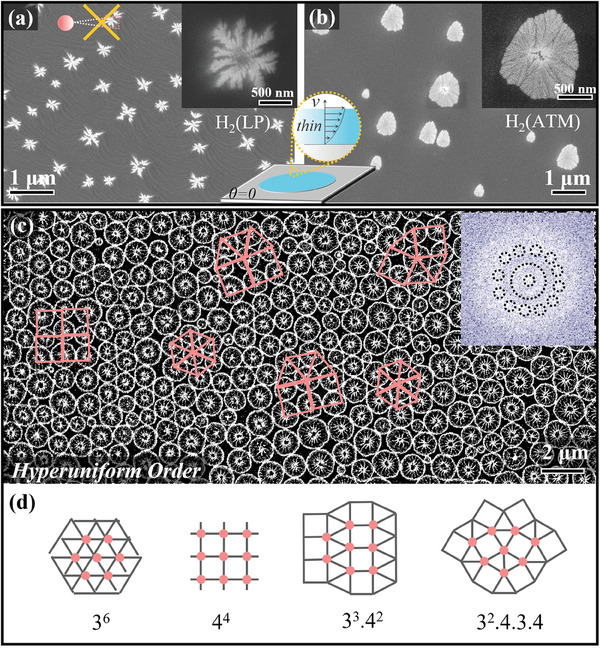
Comparative experiment and hyperuniform order. a,b) the Cu droplet becomes a wet film with introduced H_2_, and the SiO_2_ nanoparticles aggregate again to form a fractal structure (DLA or DBM), inset is the schematic diagram of the flow velocity *v* of Cu thin liquid film layer c) hyperuniform order of spoke patterns, patterns exhibit the maximally random jammed packing which obeys the Archimedean tiling, inset is fast‐Fourier‐transform (FFT) pattern of (c). d) 2D‐Archimedean tiling rule.

To verify that the local surface tension gradient of SiO_2_ nanoparticles on liquid Cu plays a key role in the interaction of complex pattern emergence, we conducted comparative experimental verification. As shown in **Figure** **4**a,b, we introduced H_2_ at 1100 °C under low pressure (LP), the Cu droplets spread into a thin film, and the pre‐deposited SiO_2_ nanoparticles reformed into a fractal shape. However, the new DLA fractal differs in that its constituent particles are fused. The reason for this is that the Cu thin liquid layer destroys the flow field interacting with the surface tension‐gradient field near the nanoparticles, but the liquid bridge‐enhanced fusion between the particles still exists (Figure 4a,b inset). When H_2_ is introduced at atmosphere pressure (ATM) and 1100 °C, as shown in Figure 4b, the nanoparticle aggregation morphology transforms into another compact fractal structure: dense‐branching morphology (DBM). Therefore, this verifies the key role of the active movement of SiO_2_ nanoparticles in forming complex patterns. Besides, as shown in Figure 4c,d, the SiO_2_ nanoparticles on the liquid metal surface show the coexistence phenomenon of local giant fluctuations and hyperuniformity that was predicted in the active fluid theory article.^[^ ^12a^ ^]^ Here, the local giant fluctuations are Spoke patterns, and hyperuniformity appears as the maximally random jammed packing of patterns. By connecting lines between adjacent pattern centers, spoke patterns exhibit the maximally random jammed packing which obeys the Archimedean tiling (3^6^), (4^4^), (3^3^.4^2^), and (3^2^.4.3.4).^[^ ^23^ ^]^ Meanwhile, we further confirm this hidden hyperuniform order exists through fast Fourier‐transform (FFT) analysis. As shown in Figure 4c inset, the areas showed some diffraction spots but were not sharp. To sum up, the hydrodynamics introduced by liquid metal can not only generate complex SiO_2_ nanoparticle patterns but also stimulate hyperuniform arrangement order. It would lay the foundation for new physical properties and new applications of SiO_2_ thin films in optics and electronics.

## 3 Conclusion

In conclusion, a SiO_2_ nanoparticle changes from Brownian motion to the self‐propelled motion by introducing liquid metal with a giant surface. This method drives the system away from thermal equilibrium, causing the aggregate morphology to transform from a DLA fractal shape to a radial spoke pattern. Moreover, the liquid metal substrate also brings hyperuniform distribution of patterns, which provides a valuable experimental method for exploring the manufacturing of “hyperuniform” smart fluid materials far from equilibrium. Through comparative experiments, numerical simulations, and theoretical analysis, it was determined that the surface tension gradient field around nanoparticles and the flow field between them are key factors in forming new film morphologies. Phenomenological models of novel pattern formation have been proposed, but the exact and deeper mechanisms remain to be clarified. However, the discovery of a new model system of “SiO_2_ nanoparticles‐liquid metal” paves a new way to understand, design, and manufacture other out‐of‐equilibrium morphology 2D materials or smart materials with self‐healing and adaptive properties. For example, an interesting application is the use of liquid metal substrates to prepare 2D materials with various novel morphologies, such as graphene, h‐BN, and MoS_2_, which may lead to new physical properties and applications in the future.

## Conflict of Interest

The authors declare no conflict of interest.

## Supporting information

Supporting Information

## Data Availability

The data that support the findings of this study are available from the corresponding author upon reasonable request.
