# Supplementary material for: Unveiling Hidden Hyperuniformity: Radial Turing Pattern Formation of Marangoni‐Driven SiO2 Nanoparticles on Liquid Metal Surface
Source: Adv Sci (Weinh). 2024 Jul 29;11(36):2400163. doi: 10.1002/advs.202400163 (PMC11423183; doi:10.1002/advs.202400163)
Supplement: Supplementary file 1 — Supporting Information [file ADVS-11-2400163-s001.docx]

**Supporting Information for**

# Unveiling Hidden Hyperuniformity: Radial Turing Pattern Formation of Marangoni-Driven SiO_2_ Nanoparticles on Liquid Metal Surface

Jinjian Guo,*^[1,2,3]^ Jie Chen,^[4]^ Kang Zhao,^[5]^ Xuedong Bai,^[1,3]^ and Wenlong Wang*^[1,3]^


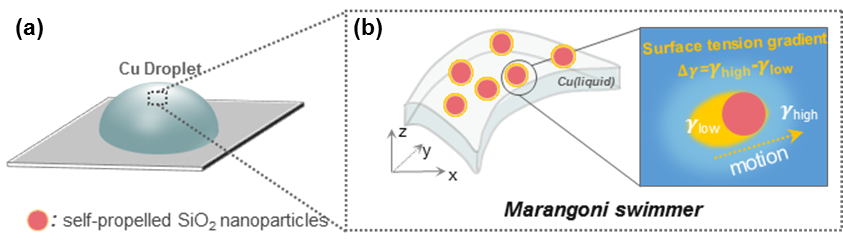
Figure S1. Particle motion: Marangoni self-propelled SiO_2_ nanoparticles

(a) SiO_2_ (O and Si element) on the liquid metal Cu, due to the weak metallicity of these elements and few free electrons, according to the surface tension electric double layer theory (1, 2), these oxides exhibit the characteristics of intrinsic high-efficiency surfactants, which can make the local surface tension of the liquid changes rapidly to almost zero (3-5). Notably, it could be estimated that the surface tension gradient of SiO_2_ nanoparticles on the liquid Cu surface is around 1488 mN/m (the surface tension of liquid Cu is 20 times that of water, about 1488 mN/m (6)). Thus, SiO_2_ nanoparticles are similar to surfactant molecules, and the adsorption on the fluid interface can change the surface tension of the fluid. As shown in (b), SiO_2_ nanoparticles behave as self-propelled Marangoni swimmers on the liquid metal surface under the Marangoni effect.

**Experiment Details:**

SiO_2_ samples were grown on Cu surfaces using the low-pressure CVD method. First, 200-mg SiO_2_ powder (≥99%, Sigma-Aldrich, particle size 0.5–10 μm) was placed in a quartz boat 10 cm near the inlet gas port of a tube-furnace (Fig. S2). Then, this system was pumped down to 0.5 Pa to remove O_2_ and heated under vacuum. During the heating process (<1,050 °C), as shown in Fig. S3, a few small amounts of SiO_2_(g) slowly volatilized from SiO_2_ powder and deposited on the Cu surface from the gas phase forming predeposited source particles [Fig. 1(a)]. This is due to the fact that even below the vaporization temperature, SiO_2_ still exists as a very small amount of gaseous SiO2 (g) (1). The predeposited source particles were observed at 1,050°C (hold 0 min) [Fig. S4(a)]. Further increasing hold time to 10 min at 1,050°C developed a SiO_2_ fractal structure on the solid substrate [Figs. 1(b) and S4(b)]. By contrast, when the solid Cu foil was heated to 1,100°C (hold 5 min), it became liquid (melting point of Cu is 1,083°C); meanwhile, the freeze spoke patterns at high temperature was obtained by rapid cooling [Figs. 1(c) and 1(d)]. In each case, the rate of heating (cooling) was 20°C/min (20°C/s). The morphology of SiO_2_ patterns was observed via scanning electron microscope (SEM, JEOL JEM-IT800, Japan; Hitachi SU8200, Japan). The composition and structure of patterns were verified via scanning transmission electron microscope/transmission electron microscope (TEM, JEOL JEM-F200, and 2010F microscope operating at 200 kV, Japan).


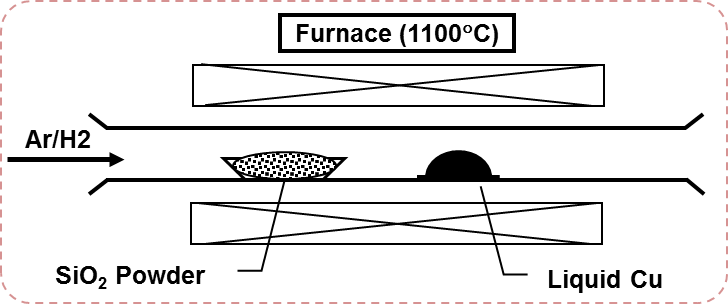


**Quartz Tube**

Figure S2. Schematic diagram of the experimental set-up.


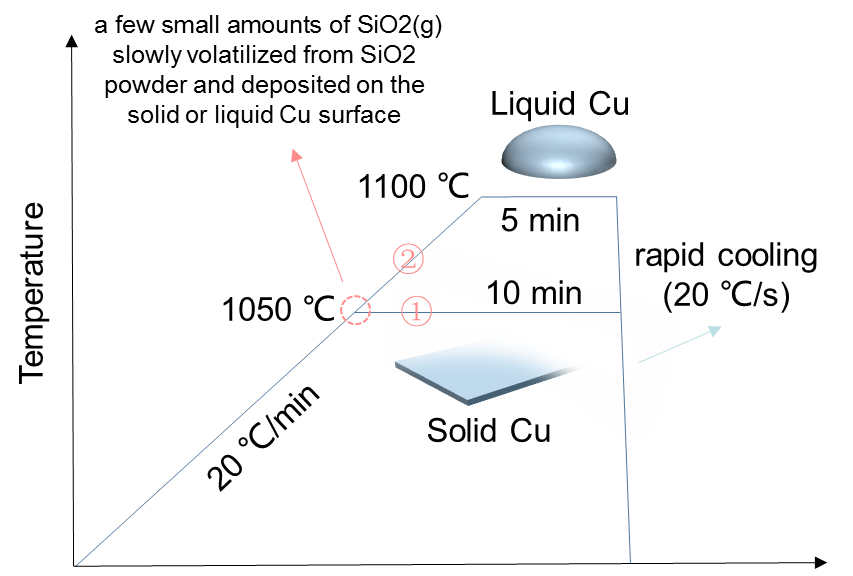


Figure S3. Schematic diagram of the experimental route.


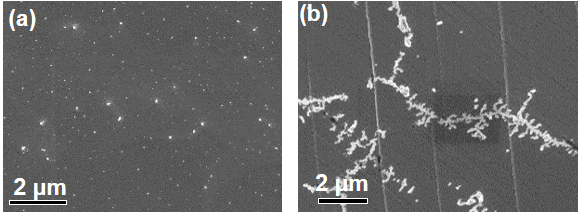


Figure S4. (a) SEM image of predeposited source SiO_2_ particles (when heated at 1,050°C for 0 min) and (b) SEM image of SiO_2_ fractal structure (when heated at 1,050°C for 10 min).


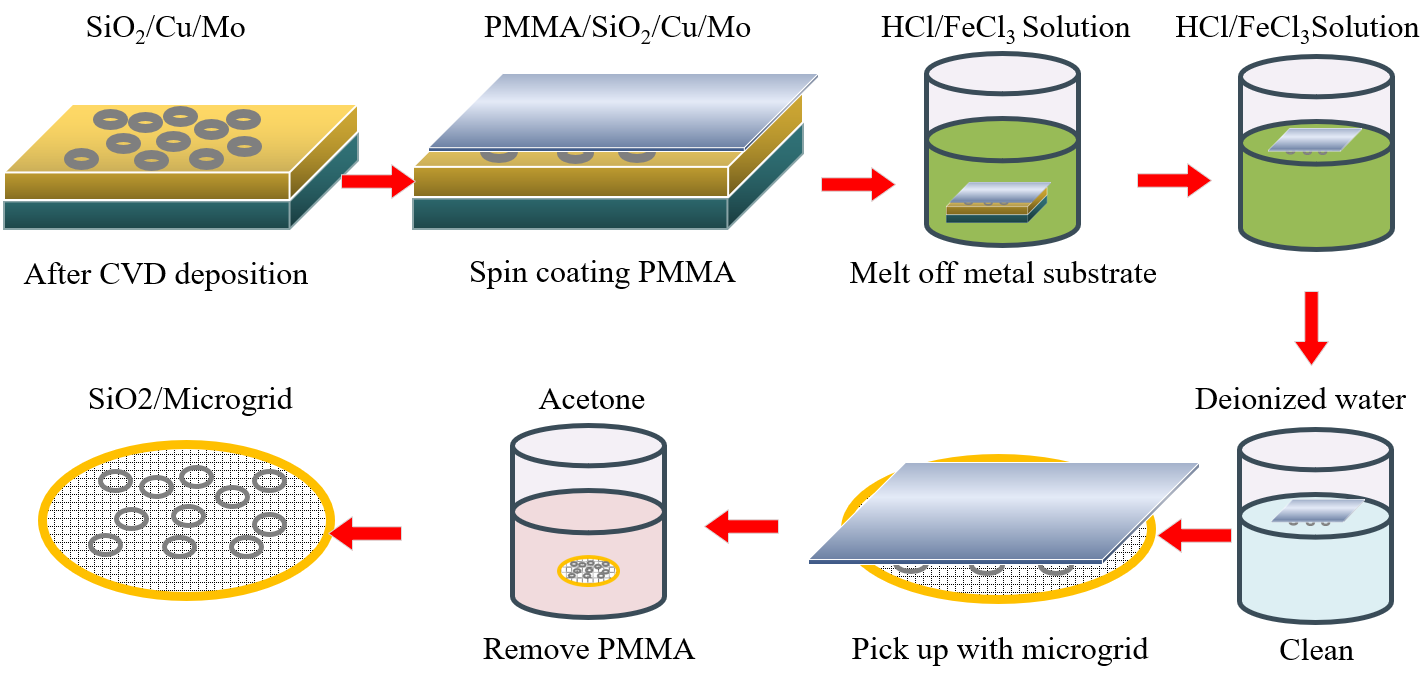


Figure S5. SiO_2_ spoke patterns grown on the copper surface were transferred onto the TEM grid by the wet chemical method using PMMA (polymethyl methacrylate, MicroChem 495, PMMA A2).

In the stepwise schematic diagram, as shown in Fig. S5, PMMA was spin-coated on sample-covered Cu substrate at 3000 rpm for 90 sec and baked at 150 °C for 1 min. After baking, the PMMA-coated sample/Cu substrate was floated in an HCl/FeCl_3_ solution for etching of the copper substrate. After etching, we scoop out the PMMA−sample membrane on a holey carbon-coated TEM grid, then clean it with deionized water and acetone to remove impurities content and dry the sample, respectively.


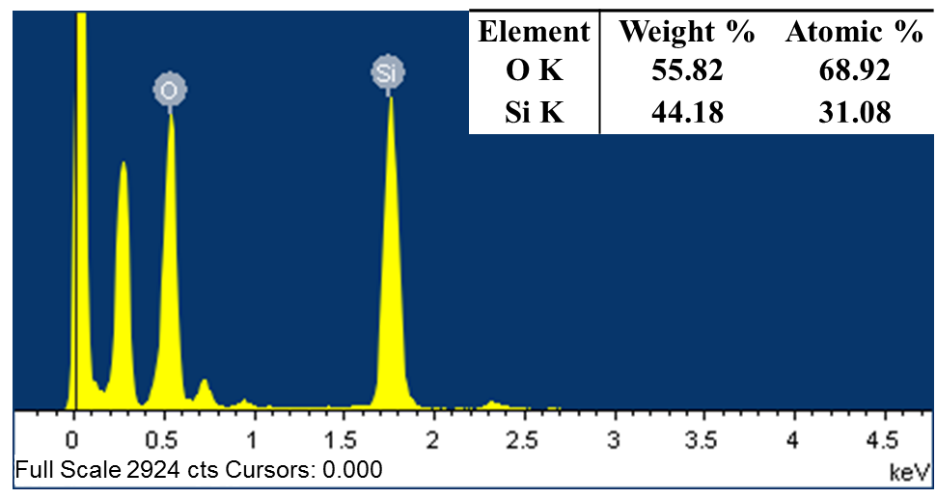


Figure S6. TEM-EDS Spectra of SiO_2_ spoke pattern. The atomic percentage of the Si and O is 68.92 % and 31.08 %, which is close to the stoichiometric ratio of SiO_2_ (2:1). The peak related to Fe was observed at 0.258 KeV, which was induced by the unwashed residual Fe element of FeCl_3_ etching solution.


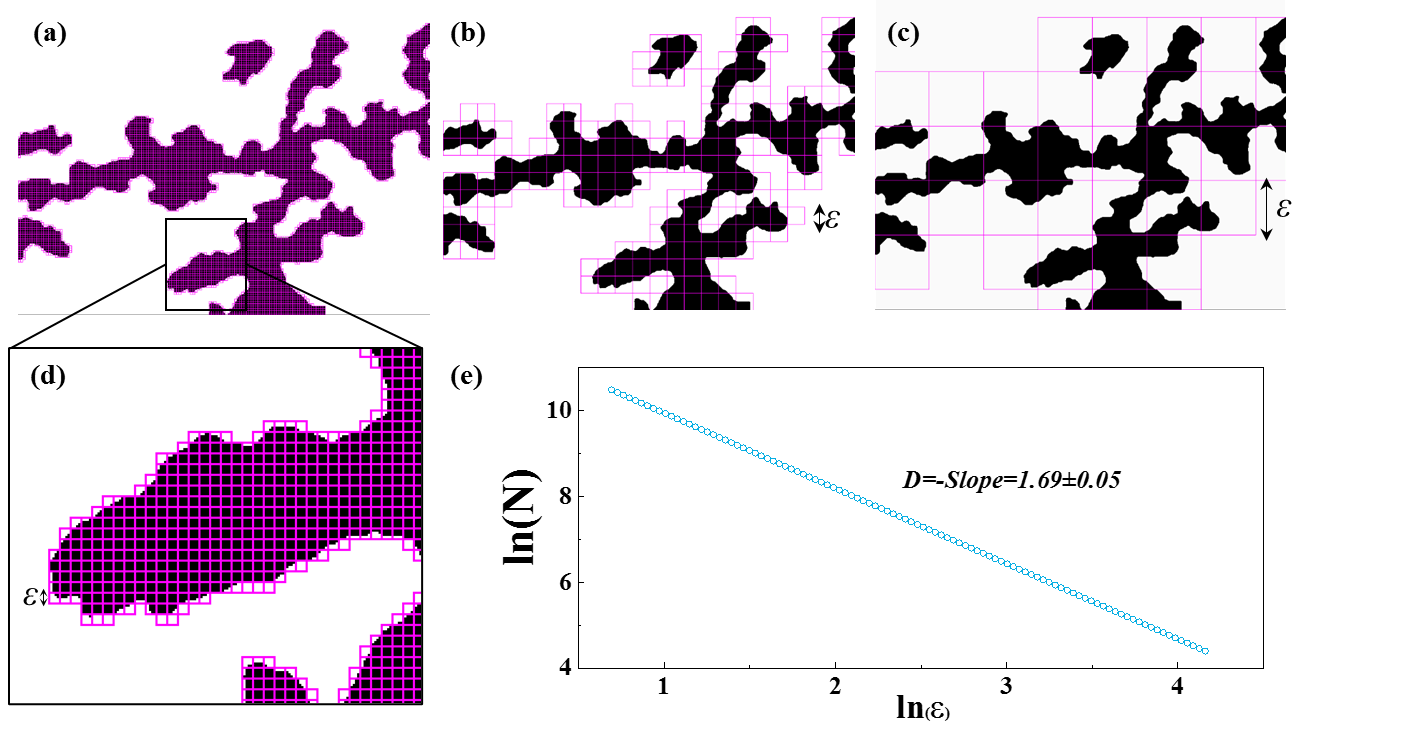


Figure S7. Fractal dimension analysis. (a-d) using different sizes of square grids (ε) to cover the image and its corresponding grid numbers (N). (e) The negative of the slope is the fractal dimension.

**References**

1. M. R. Khan, C. B. Eaker, E. F. Bowden, M. D. Dickey, Giant and switchable surface activity of liquid metal via surface oxidation. Proc. Natl. Acad. Sci. U. S. A. 111, 14047-14051 (2014).

2. R. Cade, Surface Tension as a Double-layer Phenomenon. Proceedings of the Physical Society 82, 216 (1963).

3. X. Zhao, S. Xu, J. Liu, Surface tension of liquid metal: role, mechanism and application. Frontiers in Energy, 11, 535-567 (2017).

4. B. Gallois, C. Lupis, Effect of oxygen on the surface tension of liquid copper. Metall. Trans. B 12, 549-557 (1981).

5. T. E. O'BRIEN, A. Chaklader, Effect of oxygen on the reaction between copper and sapphire. J. Am. Ceram. Soc. 57, 329-332 (1974).

6. ARSLAN H, YAVUZ M, Estimating of Surface Tension and Viscosity of Liquid Ag-Cu Alloys. Süleyman Demirel University Faculty of Arts and Science Journal of Science 16, 1, 46–65 (2021).
